# Multiparametric Prostate MRI in Biopsy-Naïve Men: A Prospective Evaluation of Performance and Biopsy Strategies

**DOI:** 10.3389/fonc.2021.745657

**Published:** 2021-10-14

**Authors:** Brage Krüger-Stokke, Helena Bertilsson, Sverre Langørgen, Torill Anita Eidhammer Sjøbakk, Tone Frost Bathen, Kirsten Margrete Selnæs

**Affiliations:** ^1^ Department of Circulation and Medical Imaging, Faculty of Medicine and Health Sciences, NTNU—Norwegian University of Science and Technology, Trondheim, Norway; ^2^ Department of Radiology and Nuclear Medicine, St. Olavs Hospital, Trondheim University Hospital, Trondheim, Norway; ^3^ Department of Cancer Research and Molecular Medicine, NTNU—Norwegian University of Science and Technology, Trondheim, Norway; ^4^ Department of Urology, St. Olavs Hospital, Trondheim University Hospital, Trondheim, Norway

**Keywords:** prostate cancer, magnetic resonance imaging, image-guided biopsy, urologic diseases, cohort studies

## Abstract

**Objectives:**

This study aims to prospectively estimate the diagnostic performance of multiparametric prostate MRI (mpMRI) and compare the detection rates of prostate cancer using cognitive targeted transrectal ultrasound (TRUS) guided biopsies, targeted MR-guided in-bore biopsies (MRGB), or both methods combined in biopsy-naïve men.

**Methods:**

The biopsy-naïve men referred for mpMRI (including T2-weighted, diffusion-weighted and dynamic contrast enhanced MRI) due to prostate cancer suspicion (elevated prostate-specific antigen or abnormal digital rectal examination) were eligible for inclusion. The images were scored according to Prostate Imaging Reporting and Data System (PI-RADS) v2, and men with PI-RADS 1–2 lesions were referred for routine systematic TRUS, while those with PI-RADS 3–5 lesions were randomized to MRGB or cognitive targeted TRUS. Men randomized to MRGB were referred to a secondary TRUS 2 weeks after MRGB. Gleason grade group ≥2 was defined as clinically significant prostate cancer. The performance of mpMRI was estimated using prostate cancer detected by any biopsy method as the reference test.

**Results:**

A total of 210 men were included. There was no suspicion of prostate cancer after mpMRI (PI-RADS 1–2) in 48% of the men. Among these, significant and insignificant prostate cancer was diagnosed in five and 11 men, respectively. Thirty-five men who scored as PI-RADS 1–2 did not undergo biopsy and were therefore excluded from the calculation of diagnostic accuracy. The overall sensitivity, specificity, negative predictive value, and positive predictive value of mpMRI for the detection of significant prostate cancer were 0.94, 0.63, 0.92, and 0.67, respectively. In patients with PI-RADS 3–5 lesions, the detection rates for significant prostate cancer were not significantly different between cognitive targeted TRUS (68.4%), MRGB (57.7%), and the combination of the two biopsy methods (64.4%). The median numbers of biopsy cores taken per patient undergoing systematic TRUS, cognitive targeted TRUS, and MRGB were 14 [8-16], 12 [6-17], and 2 [1-4] respectively.

**Conclusions:**

mpMRI, in a cohort of biopsy-naïve men, has high negative predictive value, and our results support that it is safe to avoid biopsy after negative mpMRI. Furthermore, MRGB provides a similar diagnosis to the cognitive targeted TRUS but with fewer biopsies.

## Introduction

The introduction of multiparametric prostate MRI (mpMRI) before biopsy has made an impact on how men referred to specialized healthcare services with suspicion of prostate cancer are stratified to biopsy strategies and further management (1). Due to an increasing interest in targeted biopsy (MR guided in-bore biopsy or MR/TRUS fusion biopsy), the combination of mpMRI and targeted biopsy has the potential to increase the overall accuracy in the diagnostic pathway (2).The advantages of an initial mpMRI in men with a clinical suspicion of prostate cancer are well documented (3–5), but several questions remain to be answered in regard to when and how to biopsy. The reported advantages of targeted biopsy include a more accurate characterization of tumor grade (6) and increased detection of tumors located in the anterior prostate (7). Some studies also report an overall increase in the detection of high-risk prostate cancer and decreased detection rates for low-risk prostate cancer (8–11). Moreover, in the era of multidrug-resistant microbes, the opportunity of fewer biopsy cores with targeted biopsy might contribute to a reduced risk of infection compared to systematic transrectal ultrasound-guided biopsy (TRUS) (12, 13). The advantages of targeted biopsy are favorable (14, 15); however, targeted biopsy is still not available in many small institutions, requires extensive training, and draws additional resources from an already strained system. It is therefore important not only to identify the correct biopsy method in each individual case but also to identify when and if men should be biopsied at all. Our national guidelines introduced mpMRI prior to biopsy as routine clinical practice in 2015 (16), giving the opportunity to explore the value of mpMRI as a triage tool in the prostate cancer pathway. Should biopsy-naïve men be biopsied when mpMRI is negative for prostate cancer and should they have cognitive targeted TRUS biopsies with additional systematic biopsy cores (hereafter referred to as cognitive targeted TRUS biopsy), MR-guided in-bore biopsy (MRGB), or both (combined biopsy) when mpMRI is suspicious of prostate cancer?

The objective of this study was therefore to prospectively estimate the diagnostic performance of mpMRI and compare the detection rates of prostate cancer using cognitive targeted TRUS-guided biopsies, MRGB, or both methods combined in biopsy-naïve men. The primary hypothesis was that MRGB would lead to higher detection rates for clinically significant prostate cancer compared to cognitive targeted TRUS biopsies.

## Methods

**Figure 1:**
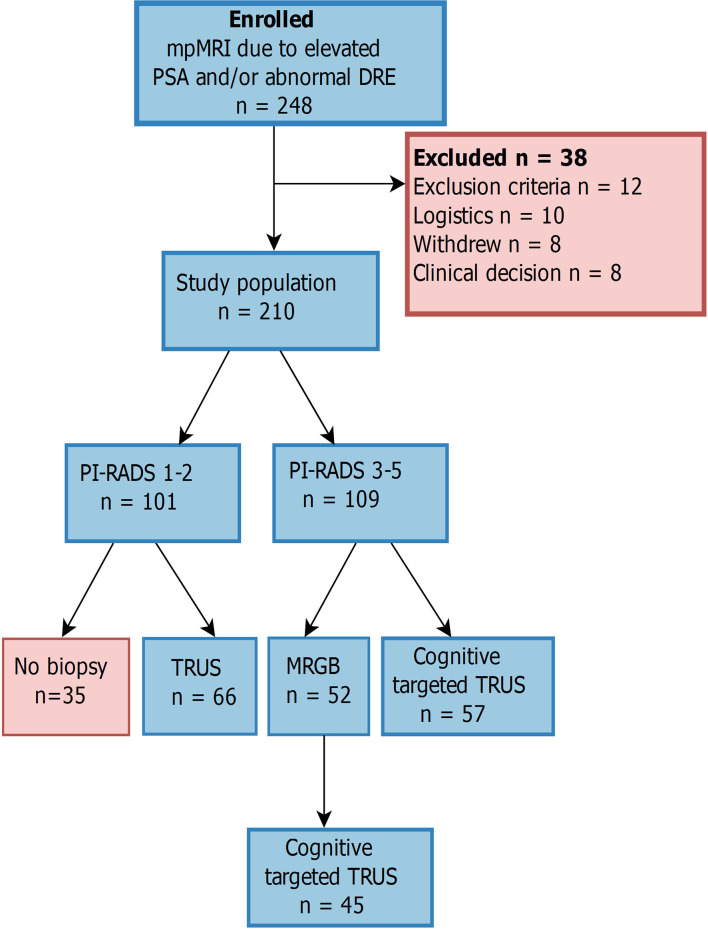
Study design. No absolute cutoff was used for elevated prostate-specific antigen, and a total of 210 men were included in the final analysis. mpMRI, multiparametric MRI; PI-RADS, Prostate Imaging Reporting and Data System; MRGB, MR-guided in-bore biopsy; TRUS, transrectal ultrasound-guided biopsy; Cognitive targeted TRUS, cognitive targeted TRUS biopsies with additional systematic biopsy cores.

This randomized prospective study was approved by our Institutional Review Board and The Regional Committee of Medical and Health Research Ethics, Central Norway (identifier REK2013/1869). Men referred according to national guidelines (16) for routine mpMRI of the prostate before TRUS from 2015 to 2017 were informed about the study, and those who gave written informed consent were enrolled (*n* = 248). The exclusion criteria were previous prostate biopsies, general contraindications to MRI, previous surgical or medical prostate treatment, and metallic implants in the pelvis or hip; 12 patients were excluded due to these. Another 10 patients were excluded due to logistic reasons, as the timing in the standardized care pathway prevented the study protocol from being conducted. Eight patients withdrew their consent, and finally, another eight patients were excluded from the study based on a clinical decision that the best follow-up for these patients were not in line with the study protocol (**Figure 1**).

### Imaging and Biopsy Pathways

All men were imaged using a 3T MRI scanner (MAGNETOM Skyra, Siemens Healthineers, Erlangen, Germany) with phased array body coil and spine coil elements for signal detection. The imaging protocol included T2-weighted imaging in three planes, axial diffusion-weighted imaging (apparent diffusion coefficient maps and calculated *b* = 1,400 s/mm^2^ images), and dynamic contrast-enhanced imaging, in accordance with the recommendations from Prostate Imaging Reporting and Data System (PI-RADS) v2 (17). Reporting was performed by one of two radiologists with 0.5 and 5 years of experience in reading prostate MRI at the onset of the study. Prior to the study, both radiologists underwent a 2-week training for reading prostate mpMRI, in accordance with PI-RADS, at a high-volume institution. PI-RADS v2 and a standardized reporting template with a maximum of three lesions were used for all reports. Men with negative imaging (PI-RADS 1–2) were referred to a urologist at our outpatient clinic for systematic TRUS. Patients with possible cancer on imaging (PI-RADS 3–5) were randomized to either cognitive targeted TRUS (with additional systematic biopsy cores) or MRGB. In order to comply with our current clinical practice, the men were referred to a secondary TRUS after MRGB. Randomization and data collection were performed by a web-based randomization and data collection system developed and administered by the Unit of Applied Clinical Research, The Faculty of Medicine and Health Sciences, Norwegian University of Science and Technology.

### Biopsies and Histopathology

Transrectal MR-guided in-bore biopsies were performed under local Xylocaine gel anesthesia by either of the two radiologists. Prior to the study, both radiologists had biopsy training during the aforementioned 2-week stay at a high-volume institution, in addition to training on a dummy model at our local institution. One or two lesions were biopsied from each patient, with two cores per lesion. The cores from MRGB were potted in separate containers corresponding to lesion identification from the standardized reporting template. TRUS biopsies were performed as part of routine clinical work at the urological department of our institution. Biopsies were systematically taken from eight regions of the prostate (left and right at four levels, one or two biopsies per region) and potted in eight separate containers. The urologists performing TRUS biopsy were not blinded to the results of the mpMRI in any of the pathways, allowing additional cognitive biopsies to be targeted towards areas suspected of harboring significant prostate cancer in men with PI-RADS 3–5 lesions. These biopsies were potted together with the systematic biopsies from the same region. TRUS biopsies from mpMRI-negative patients are referred to as systematic TRUS, while TRUS biopsies from mpMRI-positive patients are referred to as cognitive targeted TRUS. Histology was reported as Gleason score, including total biopsy length and total cancer length per container. The samples were examined by pathologists as part of routine clinical work, and we were not able to allocate the same pathologist to examine all the samples. However, all samples at our institution were double-read by two pathologists.

### Outcome Measures and Statistics

The biopsy results in this study are reported at the level of no cancer, insignificant prostate cancer, and significant prostate cancer, using Gleason grade group ≥2 (Gleason score ≥3 + 4) as the definition of significant prostate cancer (18). The overall performance of mpMRI as a triage tool is reported using significant prostate cancer detected by any biopsy method (systematic TRUS, cognitive targeted TRUS, or MRGB) as the reference test. The descriptive statistics are median (range) for continuous values and *n* (%) for categorical values, unless otherwise stated. The statistical significance of continuous values was estimated using permutation testing with 5,000 resamples. Chi-square test without Yates correction was used to compare proportions of normal and abnormal DRE in patients with PI-RADS 1–2 *versus* PI-RADS 3–5. The detection rates of insignificant prostate cancer and significant prostate cancer are reported per biopsy method, and the detection rates of MRGB and combined biopsy (both MRGB and TRUS) were compared to cognitive targeted TRUS using Pearson’s chi-square test. McNemar’s test was used to compare the outcome in men who had both MRGB and cognitive targeted TRUS. All statistics were performed using R (https://www.R-project.org/) (19).

## Results

### Clinical Characteristics and Overall Performance of mpMRI

Our final study population consisted of 210 men with median age of 65.4 years and prostate-specific antigen (PSA) of 7.12 ng/ml. There was no significant difference in age (*p* = 0.89), PSA (*p* = 0.63), prostate volume (p = 0.08), PSA density (PSAD) (*p* = 0.75), or index lesion size (*p* = 0.37) between the two randomized groups. Men with PI-RADS 3–5 lesions had significantly higher PSA (*p* < 0.001) and PSAD (*p* < 0.001) and lower prostate volume (*p* < 0.001) than those with PI-RADS 1–2 lesions (**Table 1**). Information about digital rectal examination was available in 189 (90%) men, and among these, more men with PI-RADS lesion 3–5 had abnormal digital rectal exam compared to men with PI-RADS 1–2 (*n* = 60 *vs*. *n* = 11, *p* < 0.0001). Among men categorized as PI-RADS 1–2, 35 did not undergo biopsy and are therefore excluded from the calculations of diagnostic accuracy. Overall, 78 men (44.6% of men undergoing biopsy) were diagnosed with significant prostate cancer after biopsy. Using significant prostate cancer detected by any biopsy method (systematic TRUS, cognitive targeted TRUS, or MRGB) as the reference test, the overall sensitivity, specificity, negative predictive value (NPV), and positive predictive value of mpMRI for the detection of significant prostate cancer were 0.94, 0.63, 0.92, and 0.67, respectively.

**Table 1:** Clinical characteristics of the study population.

	All	PI-RADS 1–2	PI-RADS 3–5
***n***	210	101	109
**Age (years)**	65.4 (44.1–76.4)	64.7 (45.4–75.7)	66.0 (44.1–76.4)
**PSA (ng/ml)**	7.1 (0.8–224.4)	5.8 (0.8–15.5)	9.7 (1.1–224.4)
**PSAD (ng/ml/cc)**	0.13 (0.02–10.69)	0.09 (0.02–0.44)	0.23 (0.03–10.69)
**Prostate volume (cc)**	48.0 (15.0–203.0)	57.0 (15.0–203)	40.0 (18–117)
**DRE, *n* (%)**			
Normal	118 (56.2)	78 (77.2)	40 (36.7)
Abnormal	71 (33.8)	11 (10.9)	60 (55.0)
NA	21 (10.0)	12 (11.9)	9 (8.3)
**Prostate cancer, *n* (%)** **^a^**			
No cancer	72 (41.1)	50 (75.8)	22 (20.2)
Insignificant	25 (14.3)	11 (16.6)	14 (12.8)
Significant	78 (44.6)	5 (7.5)	73 (67.0)
PI-RADS, *n* (%)			
1–2	101 (48.1)	101 (100)	0 (0.0)
3	19 (9.0)		19 (17.4)
4	27 (12.9)		27 (24.8)
5	63 (30.0)		63 (57.8)
**Index lesion size (mm)**	17.0 (6.0–44.0)		17.0 (6.0–44.0)

The descriptive statistics are median (range) for continuous values and n (%) for categorical values. Prostate cancer represents outcome after any biopsy.

PSA, prostate-specific antigen; PSAD, prostate-specific antigen density; DRE, digital rectal examination; PI-RADS, Prostate Imaging Reporting and Data System; NA, not available.

a Thirty-five men that scored as PI-RADS 1–2 had no biopsy and are not included in the number and percentage of prostate cancer cases.

### Diagnostic Accuracy of mpMRI in PI-RADS 1–2 Patients

Imaging was negative for prostate cancer (PI-RADS 1–2) in 101 men, 66 of whom had systematic TRUS with a median (range) of 14 (8–16) cores as part of their routine clinical workup. For various reasons, such as decreasing PSA level or comorbidity, systematic TRUS was deferred or canceled in 35 patients with negative imaging, and these patients were excluded from the calculation of overall diagnostic accuracy. In total, significant prostate cancer was diagnosed with systematic TRUS in five out of 66 men who underwent biopsy after negative imaging, while insignificant cancer was detected in 11 men with negative mpMRI (**Table 2**). In the five men diagnosed with significant prostate cancer, the Gleason grade groups were 2, 3, and 4 for three, one, and one man, respectively.

**Table 2:** Clinical characteristics of men with negative multiparametric prostate MRI (PI-RADS 1–2).

	No cancer	insPCa	sPCa
***n***	50	11	5
**Age (years)**	62.6 (53.2–74.0)	64.7 (55.1–74.1)	67.5 (64.3–72.0)
**PSA (ng/ml)**	6.3 (0.8–15.5)	6.2 (4.2–10.0)	7.4 (5.5–10.1)
**PSAD (ng/ml/cc)**	0.1 (0.03–0.32)	0.10 (0.04–0.4)	0.20 (0.1–0.44)
**Prostate volume (cc)**	61.5 (31.0–203.0)	54.0 (25.0–169.0)	39.0 (23.0–57.0)
**DRE, *n* (%)**			
Normal	37 (74)	10 (90.9)	3 (60.0)
Abnormal	6 (12)	0 (0.0)	2 (40.0)
NA	7 (14)	1 (9.1)	0 (0.0)

The descriptive statistics are median (range) for continuous values and n (%) for categorical values.

insPCa, insignificant prostate cancer; sPCa, significant prostate cancer; PSA, prostate-specific antigen; PSAD, prostate-specific antigen density; DRE, digital rectal examination; NA, not available.

### Diagnostic Accuracy of mpMRI in PI-RADS 3–5 Patients

**Figure 2:**
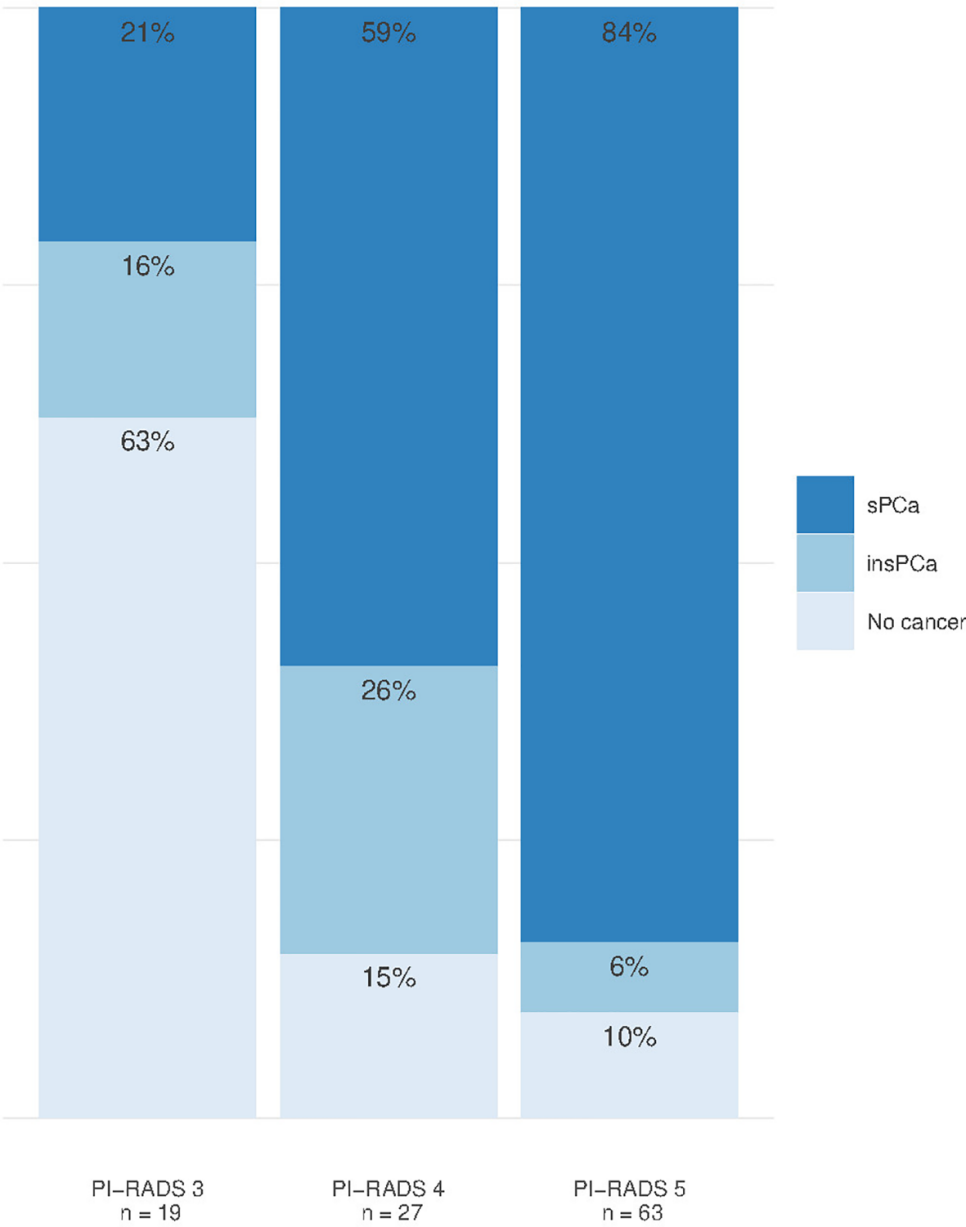
Outcome after any biopsy in men with positive mpMRI (PI-RADS 3–5). sPCa, significant prostate cancer; insPCa, insignificant prostate cancer.

We identified a total of 159 PI-RADS 3–5 lesions in 109 men, and the majority of these (65%) had one lesion, while two and three lesions were identified in 23 and 11% of the patients, respectively. Most lesions were in the peripheral zone (*n* = 115, 72%), but there were also lesions in the transition zone (*n* = 25, 16%), central zone (*n* = 3, 2%), and anterior stroma (*n* = 13, 8%). For *n* = 3 lesions, no specific region was assigned due to extensive growth over several regions and levels. Significant prostate cancer was diagnosed with MRGB and/or cognitive targeted TRUS in 73 (67%) men. In the remaining 36 men, 14 (12.8%) had insignificant prostate cancer, and 22 (20.2%) had no cancer (**Table 3**). Age (*p* < 0.006), PSA (*p* < 0.001), and PSAD (*p* < 0.001) were significantly higher in the 73 men diagnosed with significant prostate cancer. The most common PI-RADS score overall was 5 (57.8%), and significant prostate cancer was detected in 4/19 (21.1%), 16/27 (59.3%), and 53/63 (84.1%) of PI-RADS 3, 4, and 5 lesions, respectively (**Figure 2**).

**Table 3:** Clinical characteristics of men with positive multiparametric prostate MRI (PI-RADS 3–5).

	No cancer	insPCa	sPCa
***N***	22	14	73
**Age (years)**	64.4 (50.0–74.5)	62.7 (44.1–70.5)	67.1 (48.3–76.4)
**PSA (ng/ml)**	6.1 (1.1–84.7)	7.4 (3.1–24.1)	13.4 (3.7–224.4)
**PSAD (ng/ml/cc)**	0.12 (0.03–2.5)	0.13 (0.08–0.57)	0.30 (0.08–10.7)
**Prostate volume (cc)**	37.5 (23.0–100.0)	42.5 (30.0–74.0)	39.0 (18.0–117.0)
**DRE (%)**			
Normal	12 (54.5)	9 (64.3)	19 (26.0)
Abnormal	8 (36.4)	1 (7.1)	51 (69.9)
NA	2 (9.1)	4 (28.6)	3 (4.1)
PI-RADS (%)			
3	12 (54.5)	3 (21.4)	4 (5.5)
4	4 (18.2)	7 (50.0)	16 (21.9)
5	6 (27.3)	4 (28.6)	53 (72.6)

The descriptive statistics are median (range) for continuous values and n (%) for categorical values. Outcome after any biopsy method in men with positive mpMRI.

insPCa, insignificant prostate cancer; sPCa, significant prostate cancer; PSA, prostate-specific antigen; PSAD, prostate-specific antigen density; DRE, digital rectal examination; PI-RADS, Prostate Imaging Reporting and Data System; NA, not available.

### Biopsy Results of Patients Randomized to Cognitive Targeted TRUS

A total of 57 men with PI-RADS score 3–5 were randomized to cognitive targeted TRUS (**Figure 1**), and the median (range) time to biopsy after imaging was 7 (1–42) days for this cohort. Significant prostate cancer and insignificant prostate cancer were diagnosed in 39 (68.4%) and five (8.8%) men, respectively. Significant prostate cancer was diagnosed in 38 (66.6%) men with PI-RADS 4–5 lesions and only one man with a PI-RADS 3 lesion. Insignificant prostate cancer was only diagnosed in men with PI-RADS 4–5 lesions. The median (range) number of cores taken per man was 12 (6–17).

### Biopsy Results of Patients Randomized to MRGB

A total of 52 men with PI-RADS score 3–5 were randomized to MRGB, and the median (range) time to biopsy after imaging was 6.5 (1–22) days for this cohort. Significant and insignificant prostate cancer was diagnosed in 30 (57.7%) and 10 (19.2%) men, respectively. Similar to cognitive targeted TRUS, only one man with PI-RADS 3 was diagnosed with significant prostate cancer, while the remaining 29 (55.8%) had PI-RADS 4–5 lesions. Insignificant prostate cancer was diagnosed in three (5.8%) men with PI-RADS 3 lesions and seven (13.5%) men with PI-RADS 4–5 lesions. The median (range) of cores taken per man was two (1–4).

### Paired Comparison of MRGB and Subsequent TRUS

Following MRGB, 45 out of 52 men underwent a subsequent cognitive targeted TRUS biopsy. The remaining seven men did not undergo a secondary TRUS due to comorbidities or ineligibility for surgery. The median (range) time to TRUS from mpMRI was 14 (9–31) days in this cohort, and the initial MRGB had detected significant and insignificant prostate cancer in 25 (55.6%) and nine (20.0%) of these men, respectively. The secondary cognitive targeted TRUS detected additional four significant prostate cancers (8.9% increase) and two insignificant prostate cancers (4.4% increase). The secondary TRUS detected significant prostate cancer in three men previously diagnosed with insignificant prostate cancer by MRGB and in one man with previous negative MRGB. On the other hand, the secondary TRUS was negative in three men diagnosed with significant prostate cancer by MRGB and in one man diagnosed with insignificant prostate cancer by MRGB. A cross-tabulation of the results is shown in **Table 4**. When directly comparing discordant pairs in this cohort, there was no significant difference in the detection rate of significant prostate cancer (*p* = 1) or insignificant prostate cancer (*p* = 1) between MRGB and the secondary TRUS.

**Table 4:** Cross-tabulation of biopsy outcome in men with MRGB and subsequent TRUS.

	Cognitive targeted TRUS			
MRGB	No cancer	Insignificant	Significant	Total
No cancer	8	2	1	11
Insignificant	1	5	3	9
Significant	3	2	20	25
Total	12	9	24	45

Cross-tabulation of biopsy outcome in men (n = 45) biopsied with MRGB and subsequent cognitive targeted TRUS. Significant cancer defined as Gleason grade group ≥ 2.

MRGB, MR-guided in-bore biopsy.

### Summary Comparison of Pathways

**Figure 3:**
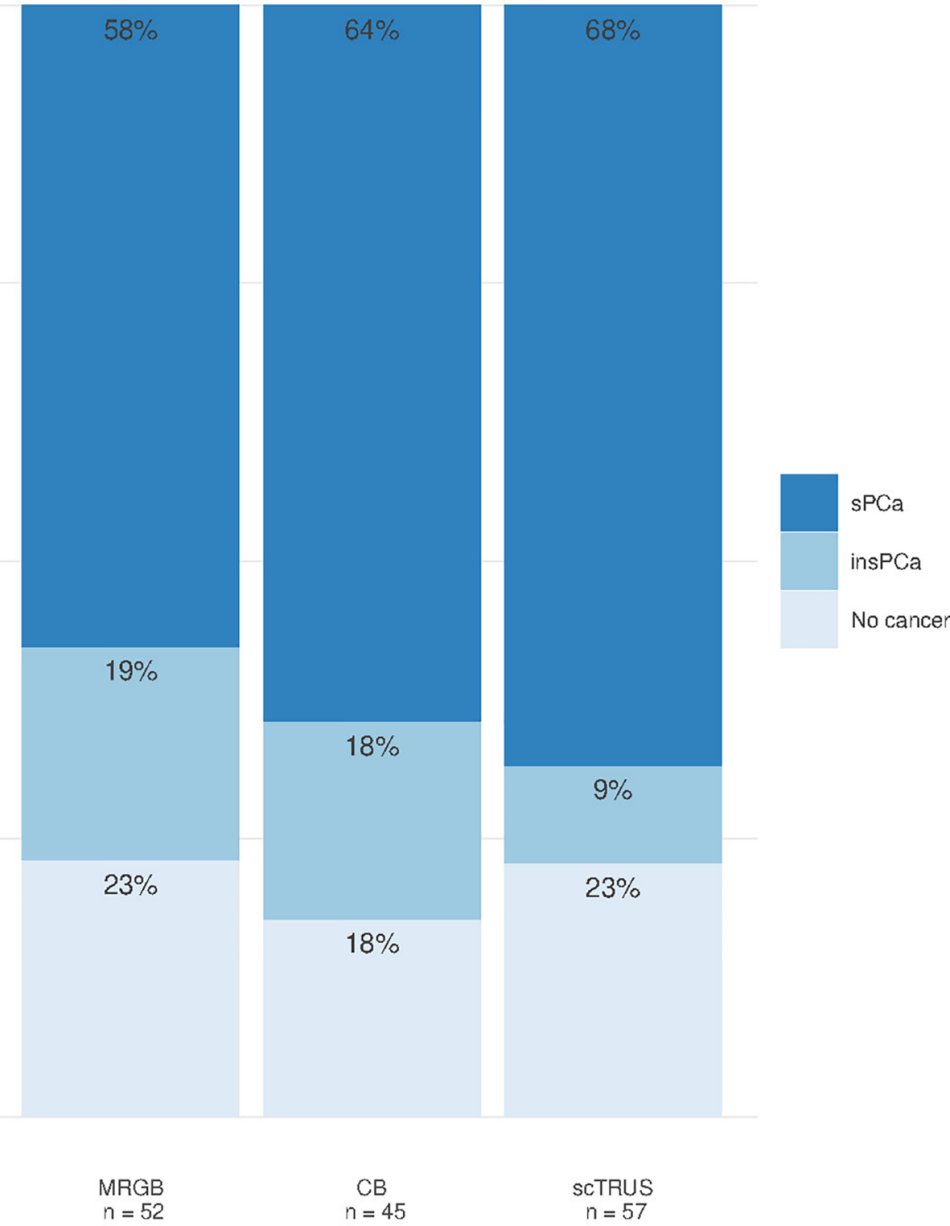
Detection rates of prostate cancer for MRGB, combined biopsy, and cognitive targeted TRUS. MRGB, MR-guided in-bore biopsy. CB, combined biopsy (MRGB with subsequent cognitive targeted TRUS with additional systematic biopsy cores); scTRUS, cognitive targeted transrectal ultrasound-guided biopsy with additional systematic biopsy cores; sPCa, significant prostate cancer; insPCa, insignificant prostate cancer.

There was no statistically significant difference in significant prostate cancer detected by cognitive targeted TRUS (68.4%) and MRGB (57.7%) or the combination of MRGB and cognitive targeted TRUS (64.4%) (*p* = 0.34 and 0.83, respectively) (**Figure 3**). Furthermore, there was no significant difference in diagnosed insignificant prostate cancer between cognitive targeted TRUS and MRGB (*p* = 0.2) or the combination of MRGB and cognitive targeted TRUS (*p* = 0.29). The overall performance of each biopsy method stratified by PI-RADS is detailed in **Table 5**.

**Table 5:** Cancer detection rates for biopsy methods stratified by PI-RADS.

		PI-RADS		Total
**Cognitive targeted TRUS**	3	4	5	
**No cancer**	7 (87.5%)	2 (16.7%)	4 (10.8%)	13 (22.8%)
**Insignificant**	0 (0.0%)	4 (33.3%)	1 (2.7%)	5 (8.8%)
**Significant**	1 (12.5%)	6 (50.0%)	32 (86.5%)	39 (68.4%)
**Total**	8 (100.0%)	12 (100.0%)	37 (100.0%)	57 (100.0%)
**MRGB**				
**No cancer**	7 (63.6%)	2 (13.3%)	3 (11.5%)	12 (23.1%)
**Insignificant**	3 (27.3%)	4 (26.7%)	3 (11.5%)	10 (19.2%)
**Significant**	1 (9.1%)	9 (60.0%)	20 (76.9%)	30 (57.7%)
**Total**	11 (100.0%)	15 (100.0%)	26 (100.0%)	52 (100.0%)
**Combined biopsy**				
**No cancer**	4 (40.0%)	2 (13.3%)	2 (10.0%)	8 (17.8%)
**Insignificant**	3 (30.0%)	3 (20.0%)	2 (10.0%)	8 (17.8%)
**Significant**	3 (30.0%)	10 (66.7%)	16 (80.0%)	29 (64.4%)
**Total**	10 (100.0%)	15 (100.0%)	20 (100.0%)	45 (100.0%)

Significant cancer was defined as Gleason grade group ≥2.

PI-RADS, Prostate Imaging Reporting and Data System; MRGB, MR-guided in-bore biopsy; Combined biopsy, patients undergoing MRGB and subsequent cognitive targeted TRUS.

## Discussion

This study prospectively included 210 biopsy-naïve men with suspicion of prostate cancer based on elevated PSA or abnormal digital rectal examination. Pre-biopsy multiparametric prostate MRI identified significant prostate cancer in 73/109 (67%) men, while there was missing significant prostate cancer in only 5/66 men (7.6% of men undergoing biopsy after negative mpMRI). Compared to cognitive targeted TRUS, we found no significant difference in the detection rates of significant prostate cancer or insignificant prostate cancer by MRGB or the combination of MRGB and a secondary cognitive targeted TRUS.

While earlier studies provided details on the ability of mpMRI to detect and characterize prostate cancer (20, 21), recent interest has shifted towards using mpMRI as a triage tool at the beginning of the prostate cancer pathway (1). Our findings from pre-biopsy mpMRI align with the results of similar studies (3, 22), adding to the evidence in favor of pre-biopsy imaging. Furthermore, our distribution of PI-RADS scores is similar to those of large-volume centers (23), strongly indicating that, given proper training, mpMRI of the prostate should be introduced at institutions with a smaller patient basis.

Despite the high NPV of mpMRI, opting out of biopsy after negative imaging remains controversial (24–26). In our cohort, 66 patients with negative mpMRI underwent biopsy. By avoiding biopsy after negative imaging, we could have avoided biopsy in these 66 men with the added benefit of not detecting insignificant prostate cancer in 11 and at the cost of missing significant prostate cancer in only five. Our NPV of 92% for significant prostate cancer is at the high end of reported values (80.4–92%) (22) but could be artificially inflated due to our imperfect reference test. In the PROMIS study by Ahmed et al., the reported NPV of mpMRI was 76% for Gleason grade group 2 prostate cancer compared to template mapping biopsy (3), and while this implies missing significant prostate cancer in ~25% of men if opting out of biopsy, diagnosis-free survival after negative mpMRI in biopsy-naïve men has been reported to be 95% after 48 months (27).

The NPV of mpMRI could be improved by establishing risk prediction models utilizing the information from mpMRI in combination with clinical variables, such as prostate volume, PSAD, and digital rectal exam (28). In our cohort, by avoiding biopsy after negative mpMRI only in patients with PSAD <0.15 ng/ml/cc and without abnormal DRE, no significant cancers would have been missed, with the additional benefit of not detecting insignificant prostate cancer in 10 and avoiding biopsy in 41 out of 66 men. The recently updated NICE guidelines on prostate cancer advocate omitting prostate biopsy after a negative mpMRI (29), and our results support this notion. Depending on life expectancy and considering the general post-biopsy complications and increasing incidence of biopsy-related infections (10, 30), we believe that avoiding biopsy after a negative mpMRI is a viable option.

There was no significant difference in cancer (significant or insignificant) detection between the different biopsy pathways in this study. This contrasts the general notion that targeted biopsy detects more significant prostate cancer and less insignificant prostate cancer (8, 9, 22, 31, 32), but in a head-to-head comparison of in-bore MRGB and systematic TRUS (blinded to mpMRI) by van der Leest et al., there was no significant difference in the detection rate of significant prostate cancer between MRGB and systematic TRUS (relative sensitivity 1.09, *p* = 0.17) but still significantly less insignificant prostate cancer detected by MRGB (relative sensitivity 0.20, *p* < 0.0001) (23). One possible explanation for the higher detection rate of significant prostate cancer with TRUS in our study could be that the urologists were not blinded to the results of mpMRI, and biopsies were performed as a combination of systematic and cognitive targeted biopsy. As prostate mpMRI was introduced as routine clinical practice by the Norwegian standardized care pathway in 2015, it would have been unethical to withhold the information from mpMRI. Furthermore, our ability to detect a difference might have been restricted by the limited number of men included in this study.

While our results imply that biopsy-naïve men with PI-RADS 3–5 lesions might not need targeted biopsy, targeted biopsies have, in line with recommendations from the PI-RADS steering committee (1), established themselves as useful tools at our institution in the setting of previously negative systematic TRUS biopsy and PI-RADS 3–5 lesions on mpMRI. In this setting, the complementary value of targeted biopsy (7) could improve the quality of life through a reduction in overdiagnosis and overtreatment while providing a safety net for men with false-negative systematic TRUS biopsy. The rising incidence of biopsy-related infections should encourage us to consider alternative biopsy methods, and while data is limited, MRGB (or transperineal biopsy) appears to be associated with a lower risk of infectious complications (13). In our study, MRGB provided a similar diagnosis to the cognitive targeted TRUS but with the advantage of obtaining significantly fewer biopsies.

A limitation of this study is the lack of a factual reference standard such as template mapping biopsy or histology from prostatectomy, preventing us from calculating the true accuracy of mpMRI and the different biopsy methods. Another limitation is that cognitive targeted cores were potted together with systematic biopsy cores from a given region. We were therefore not able to report cancer detection rates from targeted biopsy and systemic biopsy separately in the group undergoing cognitive targeted TRUS.

To summarize, in this prospective evaluation of mpMRI as a triage tool in the prostate cancer pathway, we have established that avoiding biopsy after a negative mpMRI is a viable option. Furthermore, we show that MRGB provides a similar diagnosis to the cognitive targeted TRUS but with fewer biopsies.

## Data Availability Statement

The raw data supporting the conclusions of this article will be made available by the authors, without undue reservation.

## Ethics Statement

The studies involving human participants were reviewed and approved by the Institutional Review Board and the Regional Committee of Medical and Health Research Ethics, Central Norway (identifier REK2013/1869). The patients/participants provided their written informed consent to participate in this study.

## Author Contributions

BK-S, HB, SL, TB, and KS conceptualized and designed the study. BK-S, HB, SL, TS, and KS acquired the data. KS, BK-S, and TB analyzed and interpreted the data. KS and BK-S drafted the manuscript. All authors critically revised and approved the final version of the manuscript.

## Funding

This study has received funding from the Norwegian Cancer Society (grant number 100792), St. Olavs Hospital, Trondheim University Hospital, NTNU—Norwegian University of Science and Technology, and from the joint research committee (FFU) between St. Olavs Hospital HF and the Faculty of Medicine, NTNU.

## Conflict of Interest

The authors declare that the research was conducted in the absence of any commercial or financial relationships that could be construed as a potential conflict of interest.

## Publisher’s Note

All claims expressed in this article are solely those of the authors and do not necessarily represent those of their affiliated organizations, or those of the publisher, the editors and the reviewers. Any product that may be evaluated in this article, or claim that may be made by its manufacturer, is not guaranteed or endorsed by the publisher.

## Abbreviations

DRE, digital rectal examination; mpMRI, multiparametric MRI; MRGB, MR-guided in-bore biopsy; PI-RADS, Prostate Imaging Reporting and Data System; PSA, prostate-specific antigen; PSAD, prostate-specific antigen density; TRUS, transrectal ultrasound-guided biopsy; Targeted biopsy, MR-guided in-bore biopsy or MR/TRUS-fusion biopsy; NPV, negative predictive value; PPV, positive predictive value
